# Beer Polyphenols and Menopause: Effects and Mechanisms—A Review of Current Knowledge

**DOI:** 10.1155/2017/4749131

**Published:** 2017-08-17

**Authors:** Berner Andrée Sandoval-Ramírez, Rosa M. Lamuela-Raventós, Ramon Estruch, Gemma Sasot, Monica Doménech, Anna Tresserra-Rimbau

**Affiliations:** ^1^Department of Nutrition, Food Science and Gastronomy, XaRTA, INSA, School of Pharmacy and Food Sciences, University of Barcelona, Barcelona, Spain; ^2^CIBER Fisiopatología de la Obesidad y Nutrición (CIBEROBN), Instituto de Salud Carlos III, Madrid, Spain; ^3^Hospital Clínic, Institut d'Investigacions Biomèdiques August Pi i Sunyer, University of Barcelona, Barcelona, Spain

## Abstract

Beer is one of the most frequently consumed fermented beverages in the world, and it has been part of the human diet for thousands of years. Scientific evidence obtained from the development of new techniques of food analysis over the last two decades suggests that polyphenol intake derived from moderate beer consumption may play a positive role in different health outcomes including osteoporosis and cardiovascular risk and the relief of vasomotor symptoms, which are commonly experienced during menopause and are an important reason why women seek medical care during this period; here, we review the current knowledge regarding moderate beer consumption and its possible effects on menopausal symptoms. The effect of polyphenol intake on vasomotor symptoms in menopause may be driven by the direct interaction of the phenolic compounds present in beer, such as 8-prenylnaringenin, 6-prenylnaringenin, and isoxanthohumol, with intracellular estrogen receptors that leads to the modulation of gene expression, increase in sex hormone plasma concentrations, and thus modulation of physiological hormone imbalance in menopausal women. Since traditional hormone replacement therapies increase health risks, alternative, safer treatment options are needed to alleviate menopausal symptoms in women. The present work aims to review the current data on this subject.

## 1. Introduction

Beer is one of the most frequently consumed alcoholic beverages in the world. Beer consumption ranks first in Europe, slightly above wine consumption, according to the World Health Organization [1] and third amongst alcoholic beverage preferences in North America [2]. Archaeological findings show that Chinese villagers brewed fermented alcoholic drinks as far back as 7000 BC on a small individual scale, with a production process and methods similar to those of ancient Egypt and Mesopotamia [3]. Throughout human history, products, ingredients, procedures, and techniques have evolved due to technological advances and the implementation of industrialized processes [4] further enhancing the long history of beer as a part of the human diet.

During the last two decades, scientific evidence has suggested that moderate consumption of alcoholic beverages has positive outcomes on different aspects of cardiovascular risk, as evidenced by Nogueira et al. who correlated regular daily intake of 330 ml of beer with positive changes in insulin sensitivity and lipid profiles [5]. Fermented beverages have also shown positive associations with different cardiovascular disease endpoints such as coronary heart disease, peripheral arterial disease, chronic heart failure, and stroke in which regular moderate consumption of alcohol reduced the prevalence of adverse events [6], and fermented beverages have shown anti-inflammatory properties [7]; these findings may explain the benefits of regular and moderate alcohol intake on cardiovascular disease risk [8–11]. In the last decade, the development of new techniques for food analysis has allowed the quantification of phenolic profiles [12], which, in turn, has led to new studies suggesting that regular polyphenol consumption might provide health benefits for menopausal and postmenopausal women, reducing vasomotor symptoms [13, 14] and osteoporosis [15].

Hop (*Humulus lupulus* L) is the ingredient used for beer making and is rich in phenolic compounds. Mass spectrometry analysis show that it contains around 14.4% of phenolic acids, flavonoids, proanthocyanidins, prenylated chalcones, and catechins [16]. Furthermore, malt provides 70%–80% of the total polyphenolic compounds found in beer [17]. It has been shown through high-performance liquid chromatography and posterior ultrasound separation that fermentation, boiling, and the amount of hop used to manufacture beer significantly influence the final polyphenol concentrations [18].

Menopause is induced by the permanent cessation of menstruation due to the end of ovarian follicular activity. This affects the physiology of women [19] and leads to a diminished production of estradiol which is correlated with the night sweats and hot flushes experienced by many menopausal women [20]. According to the Menopause Epidemiology Study, in which 4402 women were surveyed, these symptoms are one of the main reasons for women to seek medical care and over-the-counter treatments that provide some relief and improve the quality of life [21]. For the present work, we review the current knowledge found through online scientific libraries, PubMed and Scopus, regarding moderate beer consumption, polyphenol intake from beer, and their possible benefits for menopausal women.

## 2. Polyphenolic Compounds in Beer

**Figure 1:**
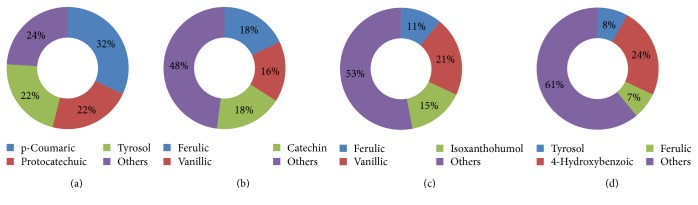
Main polyphenol content in different types of beer presented in percentages: (a) alcohol-free beer; (b) ale beer; (c) dark beer; (d) regular beer.

Beer contains amino acids, carbohydrates, vitamins, minerals, and polyphenols. As mentioned above, beer contains a diversity of polyphenols mainly derived from hops and malt [16, 22]. Moreover, during the beer fermentation process, a resin produced by hops that contains monoacyl-phlorogucinols is converted into bitter acids such as humulones and isohumulones. These molecules act as bioactive antioxidants and provide additional beneficial effects [23]. Tables 1–3 show the polyphenols found in different types of beer. Malt contains many free and total (bound) polyphenolic compounds; according to composition analysis using a liquid chromatography-antioxidant technique before and after fermentation, the concentrations of polyphenolic compounds may be increased by up to threefold after the fermentation process [24]. The main polyphenolic compounds present in beer are sinapic, ferulic, and caffeic acids. Vanillic acids are present in bound and unbound forms while 4-hydroxyphenylacetic and p-coumaric acids are present as free forms [17]. The main phenolic acids found in beer are shown in Figure 1.

## 3. Polyphenol Metabolites in Plasma

Analysis of polyphenol concentrations in plasma reveals that after ingestion, beer goes through the gastrointestinal tract. An estimated amount of between 5–10% of beer is absorbed in the small intestine, with the remaining 90–95% continuing on to the colon where it is further fermented by the gut microbiota [25], increasing the amount of polyphenols such as 4-hydroxyphenylacetic and vanillic acids absorbed [26–28]. After being absorbed, polyphenols undergo hepatic conjugation reactions with S-adenosyl methionine, sulfates, glucuronates, or a combination of them [29]. After 30 minutes, the plasma levels of nonconjugated hydroxyphenylacetic acid significantly increase. Vanillic, caffeic, and ferulic acid levels raise equally as conjugated and nonconjugated forms, with a slight prevalence of sulfate over glucuronate isoforms [30]. Composition analysis carried out in human urine samples after ingestion of wine, tea, beer, or coffee has shown that polyphenol compounds and metabolites such as resveratrol [31], 4-*O*-methylgallic acid, isoferulic acid [32], and isoxanthohumol [33] are excreted through renal filtration.  Table 4 provides detailed information about the plasma levels of polyphenol metabolites after the ingestion of beer.

## 4. Menopause: Physiology, Symptoms, and Current Treatment

Menopause is defined as the permanent cessation of menstruation as a direct result of the end of ovarian follicular activity [35]. Follicular development is a cyclical process that occurs on average every 28 days during reproductive life. However, with age, these cycles become irregular and then stop completely. This cessation causes abnormal fluctuations of sex hormones, such as the follicle-stimulating hormone (FSH), anti-Müllerian hormone, estrogen, and insulin-like growth factors-I (ILGF-I), which eventually lead to physiological and morphological changes in many organs and systems in women [36].

**Figure 2:**
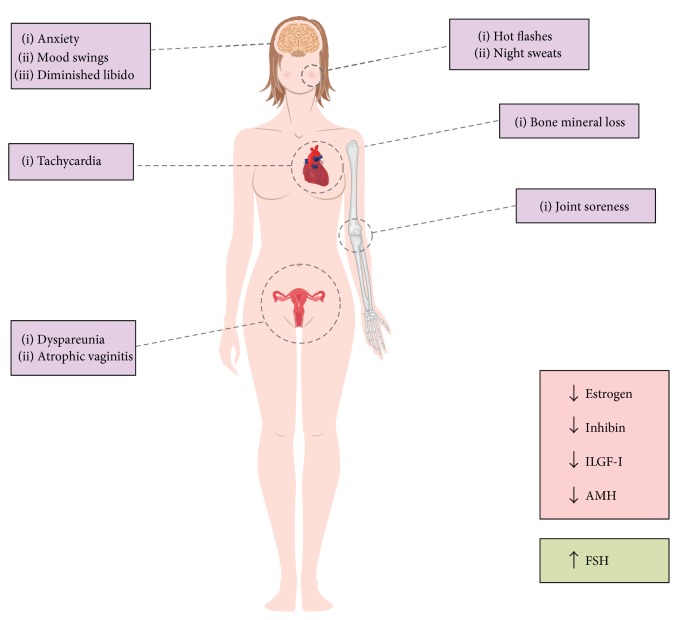
Sexual hormone status and common clinical manifestations in menopause. Insulin-like growth factor I (ILGF-I); anti-Müllerian hormone (AMH); follicle-stimulating hormone (FSH).

These physiological changes induce different symptoms and signs which are characteristic of menopausal women, such as irregular bleeding, night sweats, hot flashes, tachycardia, breast pain, lack of energy, dyspareunia, joint soreness, atrophic vaginitis, interrupted sleeping patterns, anxiety, mood swings, dry skin, and loss of libido [37, 38]. Moreover, menopause may also predispose women to a series of risks, such as an increased risk of atherosclerosis [39–43], osteopenia, and osteoporosis [44, 45] (Figure 2).

Hot flashes are one of the most frequent symptoms presented by women undergoing menopause. They have a profound impact on the quality of life and increase health costs [46]. Vasomotor symptoms represent one of the main reasons why menopausal women seek medical care and treatments in the hope of relieving their discomfort [47]. Hot flashes are the result of the brain's response to diminished and fluctuating sex hormone concentrations that occur in menopause [48, 49]. Mechanisms of temperature homeostasis on the hypothalamus and peripheral vasculature are influenced by different hormones such as ovarian hormones, norepinephrine, and serotonin. Kronenberg described the links between vasomotor symptoms and different thermal, hormonal, and autonomic parameters, demonstrating the relevance of hormones in the deregulation of core body temperature that leads to hot flashes in menopause [50].

Current menopausal treatment includes estrogen hormone replacement therapy (HRT); selective estrogen receptor modulators, such as tamoxifen and raloxifene [51]; and other medications such as selective serotonin reuptake inhibitors that alleviate vasomotor symptoms [52]. However, in different studies carried out in human patients, it has been suggested that HRT has no benefit in preventing cardiovascular disease and may even lead to an increased risk of arterial and venous thrombotic events [53], ovarian cancer [54], nonalcoholic steatohepatitis [55], and other diseases. These reports have encouraged scientists to find alternative and safer treatment options for menopausal symptoms.

## 5. Moderate Beer Intake and Health

Although it is well known that ethanol is a carcinogenic substance for humans [56], several studies have shown that regular and moderate intake of fermented beverages, such as wine and beer, may be associated with different positive health effects, such as the reduction in the risk of cardiovascular disease as evidenced by the J-shaped relation found in wine [57] and beer [58] intake on cardiovascular risk, the reduction in atheroma plaque formation [59], prevention on different cancer types [23, 60, 61], and the reduction in bone mineral loss that leads to osteoporosis and osteopenia [15, 62]. The lack of evidence attributing the same effects to spirit intake suggests that polyphenolic compounds might play an important role in the beneficial effects of moderate alcoholic beverage intake on several health outcomes [63–66].

## 6. Beer and Menopause

Several intervention studies have evaluated the effects of beer and menopause. An 8-week, randomized, double-blind, cross-over trial showed that consuming 8-prenylnaringenin (8-PN), a characteristic polyphenol from hops and beer, resulted in a significant reduction in menopause symptoms [14] and discomforts [67]. Vasomotor symptoms are believed to be caused by a slight increase in body temperature in conjunction with a smaller thermo-neutral zone [68]. These processes are controlled by a region of the anterior hypothalamus called the thermoregulatory nucleus. This area responds to sex hormones as shown by experimental models with ovariectomized rats. These rats presented significant differences in body temperature compared to a unovariectomized control group, and the differences reversed when the rats were treated with estrogens or clonidine, an alpha-adrenoceptor used for vasomotor symptom treatment, suggesting that temperature irregularities in menopause may be due to changes in the sex hormone regulatory system [69]. In the same animal model, low doses of approximately 400 *μ*g/kg/day of 8-prenylnaringenin were also able to alleviate menopausal vasomotor symptoms [70].

**Figure 3:**
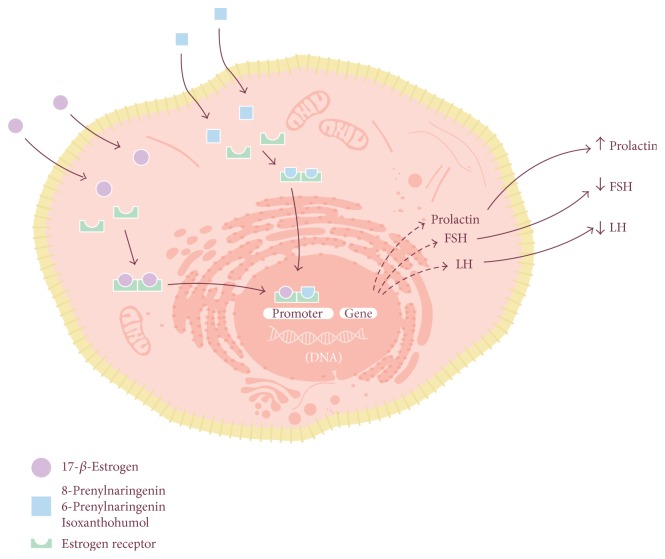
Mechanism of phytoestrogens. Like estrogen, phytoestrogen molecules travel through plasma and diffuse into the target cells, where they bind to cytoplasmatic estrogen receptors (ER). The new molecule-ER complex then dimerizes and is translocated into the nucleus, where it binds to specific promoters that decrease the expression of genes translating hormones such as follicle-stimulating hormone (FSH) and luteinizing hormone (LH) and promote the translation of prolactin. The use of estrogen receptors by 17-b-estrogen and molecules such as 8-prenylaringenin, 6-prenylaringenin, and isoxanthohumol explains why these molecules can be used to decrease the intensity of many classical symptoms of menopause.

The effect of 8-prenylnaringenin may be explained by its strong affinity for both alpha and beta estrogen receptors (ER). The binding of 8-PN and the consequent activation of ERs lead to the stimulation of alkaline phosphatase activity and upregulate the activity of progesterone receptors and presenelin-2 [14], both of which are estrogen-stimulated genes (Figure 3). In addition, low doses of 8-prenylnaringenin increase the libido of menopausal women [71].

The absorption of hop phenolic acid and the pharmacokinetics and possible health benefits of hops have been studied in women [72]; however, at present, no clinical trial has assessed the effects of moderate beer consumption on menopausal women.

## 7. Summary

Menopause is a physiological condition that causes significant discomfort in many women around the world with the presentation of a myriad of symptoms related to an imbalance in sex hormone levels. Hot flashes and night sweats are two of the most common clinical findings in menopausal women that lead them to seek medical care. Since traditional hormone replacement therapies increase health risks, alternative, safer treatment options are needed. Hop and beer polyphenols seem to be an alternative to alleviate the menopausal symptoms presented by women.

There is evidence that regular and moderate intake of the polyphenols commonly found in hop and beer may help to relieve many common symptoms presented by women undergoing menopause. Said benefits can also be obtained by menopausal women from regular alcohol-free beer consumption, since ingredients used and most processes are shared between alcohol-free and regular beer. Alcohol-free beer could provide women with all the same possible benefits, without the risk of gastrointestinal pathologies and cancer that frequent alcohol consumption represents to health. Nonetheless, randomized intervention clinical trials are needed to confirm their efficacy.

## Figures and Tables

**Table 1:** Flavonoids contained in different types of beer.

Molecule	Mean content (mg/100 ml)			
	Alcohol-free	Ale	Dark	Regular
Chalcones				
Xanthohumol	0.0003	0.0100	0.0300	0.0014
Flavanols				
Catechin	0.1000	0.3300	0.0200	0.1100
Epicatechin	0.0056	0.0500	0.0100	0.0600
Procyanidin dimer B3				0.1600
Procyanidin trimer C2				0.0300
Prodelphinidin trimer C-GC-C				0.0200
Prodelphinidin trimer GC-C-C				0.0100
Prodelphinidin trimer GC-GC-C				0.0400
Prodelphinin dimer B3				0.1800
Flavanones				
6-Geranylnaringenin		0.0011	0.0027	0.0004
6-Prenylaringenin	0.0007	0.0200	0.0200	0.0026
8-Prenylaringenin	0.0003	0.0044	0.0092	0.0010
Isoxanthohumol	0.0100	0.2100	0.1200	0.0400
Naringin				0.0008
Flavones				
Apigenin				0.0042
Flavonols				
3,7-Dimethylquercetin				0.0003
Myricetin				0.0007
Quercetin				0.0067
Quercetin 3-*O*-arabinoside				0.0006
Quercetin 3-*O*-rutinoside				0.0900
Isoflavonoids				
Biochanin A		0.0005		0.0015
Daidzein		0.0005		
Genistein		0.0010		

Data from the Phenol-Explorer database [12].

**Table 2:** Phenolic acids contained in different types of beer.

Molecule	Mean content (mg/100 ml)			
	Alcohol-free	Ale	Dark	Regular
Hydroxybenzoic acids				
2,6-Dihydroxybenzoic acid				0.0900
2-Hydroxybenzoic acid	0.0011			0.2000
3,5-Dihydroxybenzoic acid				0.0300
3-Hydroxybenzoic acid				0.0300
4-Hydroxybenzoic acid	0.0073	0.1100	0.0700	0.9600
Gallic acid		0.1100	0.0300	0.0700
Gallic 3-O-gallate				0.2600
Gentisic acid				0.0300
Protocatechuic acid	0.2700	0.0600	0.0400	0.0500
Syringic acid		0.1100		0.0200
Vanillic acid	0.0300	0.2900	0.1700	0.0700
Hydroxycinnamic acids				
4-Caffeoylquinic acid				0.0100
5-Caffeoylquinic acid				0.0800
Caffeic acid	0.0100	0.0075	0.0300	0.0300
Ferulic acid	0.1200	0.3300	0.0900	0.2600
*m*-Coumaric acid				0.0200
*o*-Coumaric acid				0.1500
*p*-Coumaric acid	0.4000	0.1200	0.0500	0.1000
Sinapic acid	0.0073	0.0700	0.0300	0.0200
Hydroxyphenylacetic acids				
4-Hydroxyphenylacetic acid				0.0300
Homovanillic acid				0.0500

Data from the Phenol-Explorer database [12].

**Table 3:** Other phenolic compounds contained in beer.

Molecule	Mean content (mg/100 ml)			
	Alcohol-free	Ale	Dark	Regular
2,3-Dihydroxy-1-guaiacylpropanone	0.0025			0.0034
3-Methylcatechol			0.0029	0.0001
4-Ethylcatechol			0.0010	0.0006
4-Hydroxycoumarin				0.1100
4-Methylcatechol			0.0022	
4-Vinylguaiacol		0.0100	0.0300	0.1500
4-Vinylphenol			0.0300	0.0045
Catechol			0.0100	0.0011
Esculin				0.0200
Pyrogallol			0.0300	0.0047
Tyrosol	0.2700			0.3200
Umbelliferone				0.0017
Vanillin	0.0048			0.0200

Data from the Phenol-Explorer database [12].

**Table 4:** Mean plasmatic levels of polyphenolic metabolites after beer intake.

Polyphenolic metabolite	Dose per day	Mean concentration (plasma)	T-Max (h)	Ref.
Ferulic acid	500 ml	0.11 *μ*mol/l	0.5	[34]
4-Hydroxyphenylacetic acid	500 ml	1.4 *μ*mol/l	0.5	[30]
Vanillic acid	500 ml	0.11 *μ*mol/l	0.5	[30]
*p*-Coumaric acid	500 ml	0.05–0.07 *μ*mol/l	0.5	[30]
Caffeic acid	500 ml	0.05–0.07 *μ*mol/l	0.5	[30]

T-Max: time when maximal concentration is achieved.
